# Exploring factors for predicting colchicine responsiveness in children with PFAPA

**DOI:** 10.1007/s00431-024-05703-3

**Published:** 2024-07-31

**Authors:** Zeynep Özaslan, Abdulvahap Şen, Sıla Atamyıldız Uçar, Mustafa Çakan, Bengisu Sanisoğlu, Feray Kaya, Gülçin Otar Yener, Ferhat Demir, Ayşe Tanatar, Semanur Özdel, Kübra Öztürk, Nihal Şahin, Hafize Emine Sönmez, Nuray Aktay Ayaz, Betül Sözeri

**Affiliations:** 1 Department of Pediatric Rheumatology, Faculty of Medicine, MD, Kocaeli University, Kocaeli, Turkey; 2 Department of Pediatric Rheumatology, Ankara Etlik City Hospital, Ankara, Turkey; 3 Department of Pediatric Rheumatology, Health Sciences University, Umraniye Training and Research Hospital, Istanbul, Turkey; 4 Department of Pediatric Rheumatology, Zeynep Kamil Training and Research Hospital, Istanbul, Turkey; 5 Department of Pediatrics, Department of Pediatric Rheumatology, İstanbul University, Istanbul, Turkey; 6 Department of Pediatric Rheumatology, İstanbul Medeniyet University, İstanbul, Turkey; 7 Department of Pediatric Rheumatology, Eskişehir City Hospital, Eskişehir, Turkey; 8 Department of Pediatric Rheumatology, Acıbadem Ataşehir Hospital, Istanbul, Turkey; 9 Department of Pediatric Rheumatology, Gaziantep City Hospital, Gaziantep, Turkey

**Keywords:** PFAPA, Colchicine, Treatment response, *MEFV*

## Abstract

Periodic fever, aphthous stomatitis, pharyngitis, and cervical adenitis syndrome (PFAPA) are the most common autoinflammatory syndromes in children. This study aimed to evaluate the clinical and laboratory parameters that may predict colchicine responsiveness.This retrospective, multicenter, cross-sectional study involved nine pediatric rheumatology centers from our country., The patients diagnosed with PFAPA were compared on the basis of their responses to colchicine. In the 806 (42.3% female 57.7% male) patients, the most common clinical findings were fever (100%), exudative tonsillitis (86.5%), pharyngitis (80.9%), and aphthous stomatitis (50.5%). The mean attack frequency was 13.5 ± 6.8 attacks per year lasting for a mean of 3.9 ± 1.1 days. Colchicine treatment was attempted in 519 (64.4%) patients, with 419 (80.7%) showing a favorable response. In patients who underwent *MEFV* gene analysis (70.8%), the most common variant was M694V heterozygous (16.8%). The presence of pharyngitis (*p* = 0.03, 95% CI 0.885 to 0.994), the presence of arthralgia (*p* = 0.04, 95% CI 0.169 to 0.958), and having more frequent attacks (*p* = 0.001, 95% CI 0.028 to 0.748) were found to be associated with colchicine unresponsiveness, whereas the carriage of the M694V variant (*p* = 0.001, 95% CI 0.065 to 0.242) was associated with colchicine responsiveness.

*Conclusion*: This study identified the presence of pharyngitis, arthralgia, and increased attack frequency in patients with PFAPA as factors predicting colchicine unresponsiveness, whereas the carriage of the M694V variant emerged as a predictor of colchicine responsiveness. Predicting colchicine response at disease onset may facilitate a more effective management of PFAPA.

**Table:** 

**What is Known**: • *Colchicine treatment can be used in the prophylaxis of PFAPA disease.* • *Having the MEFV variant is the most commonly known factor in predicting response to colchicine.*
**What is New**: • *The presence of pharyngitis or arthralgia, and more frequent attacks in PFAPA disease were found to be independently associated with colchicine unresponsiveness.* • *Carrying the M694V variant was identified as the sole factor predicting colchicine responsiveness.*

**What is Known**:

• *Colchicine treatment can be used in the prophylaxis of PFAPA disease.*

• *Having the MEFV variant is the most commonly known factor in predicting response to colchicine.*

**What is New**:

• *The presence of pharyngitis or arthralgia, and more frequent attacks in PFAPA disease were found to be independently associated with colchicine unresponsiveness.*

• *Carrying the M694V variant was identified as the sole factor predicting colchicine responsiveness.*

## Introduction

Periodic fever, aphthous stomatitis, pharyngitis, and cervical adenitis syndrome (PFAPA) are the most common autoinflammatory periodic fever syndromes in children. The precise prevalence and etiology of PFAPA are currently unknown; however, a study conducted in Scandinavia estimated the incidence to be 2.3 per 10,000 children [1]. In preschool-aged children, PFAPA is characterized by recurrent episodes of high fever lasting 3–7 days and occurring every 2–8 weeks. Diagnosis is often delayed because of the lack of specific laboratory findings for the disease, leading to variations in treatment approaches [2, 3]. A typical PFAPA episode presents with fever, pharyngitis, oral aphthous lesions, and cervical lymphadenitis. During attacks, abdominal pain, arthralgia, arthritis, headache, rash, diarrhea, and vomiting may also be present [4]. In addition, 60% of patients may experience prodromal symptoms such as fatigue. As the child grows, the frequency and duration of episodes typically decrease, and PFAPA commonly resolves within a few years [1–3].

During PFAPA episodes, patients and their families often experience heightened anxiety, leading to a notable decline in their quality of life. Treatment achieves complete remission of the disease or at least reduce disease activity, thereby positively impacting patients and families. The objective is to restore a satisfactory quality of life and promote uninterrupted child development, especially concerning normal school performance. Commonly used treatment strategies for PFAPA include antipyretics during episodes, corticosteroids for abortive treatment, colchicine or cimetidine for prophylaxis, and tonsillectomy as a surgical option [5].

Intermittent single doses of steroids can be used during PFAPA attacks [5, 6]. Nevertheless, it may increase the frequency of attacks. In such cases, the regular administration of colchicine is an alternative therapeutic approach. Colchicine, a compound derived from plants, exerts its anti-inflammatory effects by binding to tubulins, thereby impeding the assembly and polymerization of microtubules. It may be employed prophylactically to reduce the frequency of PFAPA attacks in children, with studies demonstrating a decrease in attack frequency following regular colchicine treatment [6, 7]. Understanding the factors that influence colchicine response in PFAPA syndrome is crucial for effective patient management. This study aimed to evaluate whether specific parameters can predict colchicine response by analyzing the clinical, genetic, and laboratory characteristics of patients with PFAPA.

## Methods

This study is retrospective, multicenter and cross-sectional study. Nine pediatric rheumatology referral centers from our country were enrolled in the study. The patients diagnosed with PFAPA between January 2019 and January 2024 were enrolled it. A total of 806 patients with PFAPA were included in the study. The diagnosis of PFAPA syndrome was made according to the Eurofever/PRINTO classification criteria [2]. Patients who meet the diagnosis of FMF according to Yalçınkaya and Özen criteria or meet the diagnostic criteria of any of monogenic autoinflammatory diseases were excluded.

Steroid responsiveness was determined by the resolution of the disease episode within 6 h after a single dose of steroid administration during the disease attack. Patients whose disease episodes ceased or became less frequent after colchicine treatment were classified as "colchicine responsive" [8]. The disease was considered to have ceased if the patient did not experience an attack for at least 1 year. Patients who were treated with colchicine among all PFAPA patients were divided into two groups as colchicine responsive and colchicine non-responsive.

Demographic data, laboratory findings, *Mediterranean FeVer* (*MEFV*) gene analysis, parameters such as fever episodes, associated symptoms, effectiveness of various therapies (including glucocorticoids, colchicine, tonsillectomy, and/or adenotonsillectomy), and age at the time of surgery were documented from the patient’s medical records.

Data from all centers were entered into a common Excel worksheet. Duplicate entries were removed, and the final version was transferred to SPSS software for analysis.

### Statistical analysis

Statistical analyses were conducted using the SPSS software version 21. The variables were assessed using visual (histogram and probability plots) and analytical methods (Kolmogorov–Smirnov) to determine their distribution. Descriptive analysis data are presented as mean ± standard deviation or median (minimum–maximum) where appropriate. Categorical variables were compared using the chi-square test or Fisher’s exact test, as appropriate. The Student’s T-test or Mann–Whitney U-test was used to compare continuous data between the two groups. Variables with a p-value of ≤ 0.05 in univariate analysis were entered into a logistic regression analysis to identify independent predictors of colchicine resistance. Model fit was assessed using Hosmer–Lemeshow goodness-of-fit statistics. A significance level of 5% was used. Risk scores for each selected variable were weighted based on β coefficients (‘x’ = log of the OR) in the final model.

## Results

A total of 806 patients were enrolled in the study. Of them, 341 (42.3%) were female and 465 (57.7%) were male. The median current age of the patients was 68 (17–180) months. The median age at diagnosis and symptom onset were 44 (9–140) and 24 (3–120) months, respectively.

Fever was the most common clinical finding, reported in all 806 (100%) patients, followed by exudative tonsillitis in 697 (86.5%), pharyngitis in 652 (80.9%), aphthous stomatitis in 407 (50.5%), cervical lymphadenopathy in 340 (42.2%), abdominal pain in 253 (31.4%), arthralgia in 191 (23.7%), headache in 34 (4.12%), and arthritis in 8 (1%) of the patients. The mean attack frequency was 13.5 ± 6.8 attacks per year with a mean of 3.9 ± 1.1 days. The mean interval time between attacks was 29 ± 14.6 days.

At the time of the attack, the median white blood cell count (WBC) was 12460 (6000–33540)/mm3, erythrocyte sedimentation rate (ESR) was 26 (20–120) mm/h, and C-reactive protein (CRP) was 56 (10–324) mg/dL.

Out of 659 (81.8%) patients who received steroids during attacks, 602 (91.3%) showed resolution of the febrile episode within 6 h. However, the attack frequency increased in 214 (32.4%) cases.

Colchicine treatment was attempted in 519 (64.4%) patients. Of these, 419 (80.7%) were colchicine-responsive, whereas 12 (2.3%) showed partial response. Tonsillectomy/adenoidectomy was advised in 134 (16.6%) patients and performed on 90 (11.2%) during follow-up. This resulted in the cessation of disease episodes in 74 (82.2%) patients who underwent the procedure.

MEFV gene sequencing was performed on 571 (70.8%) patients. Among them, the genetic mutation was not detected in 335 (58.1%) patients, whereas the most common variant was M694V heterozygote in 96 (16.8%) patients, followed by E148Q heterozygote in 52 (9.1%), V726A heterozygote in 20 (3.5%), and M680I heterozygote in 15 (2.6%) patients. The genetic test results are summarized in Table 1. One hundred forty (24.1%) carried exon 10 heterozygote variants, 62 (10.5%) carried non-exon 10 heterozygote variants, 9 (1.6%) carried exon 10/non-exon 10 compound heterozygote variants, 5 (0.08%) were non-exon 10 homozygous, 5 (0.08%) were non-exon 10/non-exon 10 compound heterozygotes, 3 (0.05%) were exon 10 compound heterozygotes, and 2 (0.03%) were exon 10 homozygous. The clinical and laboratory parameters of the patients were compared based on their responses to colchicine (Table 2).

**Table 1:** Genetic test results of patients (n = 571)

Genetic variants	n (%)
M694V/-	96 (16.8)
E148Q/-	53 (9.2)
V726A/-	20 (3.5)
M680I/-	15 (2.6)
K695R/-	6 (1.1)
P369S/-	5 (0.8)
E148Q/E148Q	4 (0.7)
R761H/-	3 (0.5)
R408Q/P369S	3 (0.5)
M694V/E148Q	2 (0.3)
V726A/E148Q	2 (0.3)
I591T/-	2 (0.3)
M694V/V726A	1 (0.17)
M694V/S369A	1 (0.17)
M694V/R408Q/P369S	1 (0.17)
M694V/P369S	1 (0.17)
M694V/M694V	1 (0.17)
M694V/K695R	1 (0.17)
M694V/K695R/K695R	1 (0.17)
R408Q/E148Q/P369S	1 (0.17)
P588P/P588P	1 (0.17)
M680I / A744S	1 (0.17)
F479L/-	1 (0.17)
E148Q/P369S	1 (0.17)
A744S/-	1 (0.17)
Y471X/-	1 (0.17)
V726A/R408Q	1 (0.17)
Negative	345 (60.4)
Total	571 (100)

**Table 2:** Comparison of clinical and laboratory parameters based on colchicine-responsiveness in patients with PFAPA syndrome

	Colchicine responsive (*n* = 419)	Colchicine unresponsive (*n* = 88)	*P* value
Gender (Female/Male)	180 /239	40/48	0.66
Age at symptom onset (months)	24 (3–120)	23.5 (5–72)	0.32
Age at diagnosis (months)	42 (9–140)	37.5 (15–80)	0.24
Fever, n (%)	419 (100)	88 (100)	NA
Exudative tonsillitis, n (%)	365 (87.1)	77(87.5)	0.92
Pharyngitis, n (%)	327 (78)	80 (90.9)	**0.006**
Aphthous stomatitis, n (%)	210 (50.1)	51 (58)	0.18
Cervical lymphadenopathy, n (%)	174 (41.5)	39 (44.3)	0.63
Headache, n (%)	15 (3.6)	4 (4.5)	0.66
Abdominal pain, n (%)	135 (32.3)	34 (38.6)	0.25
Arthralgia, n (%)	97 (23.2)	30 (34.1)	**0.03**
Arthritis, n (%)	3 (0.7)	2 (2.3)	0.17
Attack frequency, per year	13.6 ± 7.1	16.1 ± 7.1	**0.001**
Duration of attacks, day	4.1 ± 0.9	3.8 ± 1.1	**0.001**
The interval time between attacks, days	28.4 ± 20	28.5 ± 12.1	0.18
White blood count, mm^3^	13,000 (6000–32000)	13,205 (8500–33540)	0.44
Erythrocyte sedimentation rate, mm/hr	26.5 (20–106)	32.5 (30–100)	0.23
C reactive protein (CRP), mg/L	59.9 (10–274)	65.5 (8–256)	0.28
Steroids, n (%)	360 (85.9)	78 (86.6)	0.49
Responsive to steroids, n (%)	334 (92.5)	68 (87.2)	0.12
Increased attack frequency with steroids, n (%)	126 (30.1)	37 (42)	0.09
Tonsillectomy/adenoidectomy	15 (3.6)	36 (40.9)	** < 0.001**
*MEFV* variants			
Carrying exon 10 variants	123 (61.9)	17 (63)	0.367
Carrying M694V variants	95 (20.2)	10 (6.9)	** < 0.001**

To identify independent predictors of colchicine response, all clinical and laboratory results were analyzed by univariate analysis. Those that were significant in the univariate analysis were included in the multivariate analysis. In a multivariate regression analysis, the presence of pharyngitis (*p* = 0.03, CI95% 0.885 to 0.994), the presence of arthralgia (*p* = 0.04, CI95% 0.169 to 0.958), and more frequent attacks (*p* = 0.001, CI95% 0.028 to 0.748) were found to be independently associated with colchicine unresponsiveness, while carrying the M694V variant (*p* = 0.001, CI95% 0.065 to 0.242) was the sole factor predicting colchicine responsiveness.

## Discussion

There is no consensus on the optimal therapeutic strategy for PFAPA syndrome. The primary objective of treatment is to manage acute attacks and decrease their frequency. However, decisions regarding colchicine resistance and the potential necessity for tonsillectomy have predominantly relied on expert consensus, given the absence of an objective laboratory marker capable of interpreting and predicting the disease’s course. When determining the most suitable treatment strategy for patients with PFAPA, it is essential to carefully weigh the risks and benefits of each option and involve the family in the decision-making process. In this multicenter study involving a substantial sample size, we examined parameters for predicting the response to colchicine in patients with PFAPA, focusing on clinical and laboratory findings. Our findings indicate that the presence of pharyngitis, arthralgia, and more frequent attacks are predictive of colchicine resistance in PFAPA. In addition, we observed that the presence of the M694V variant carrier status significantly predicts colchicine sensitivity.

The demographic and clinical data of patients with PFAPA have been comprehensively examined in numerous prior studies. Consistent with previous findings, our study revealed a slight male predominance among patients with PFAPA, which was observed in both the colchicine-responsive and colchicine-resistant groups [1, 6, 9]. Various studies have consistently reported exudative pharyngitis, aphthous stomatitis, cervical lymphadenitis, and abdominal pain as the most frequently observed clinical findings in patients with PFAPA [6, 10–14]. Correspondingly, exudative tonsillitis, pharyngitis, aphthous stomatitis, and cervical lymphadenopathy were the most common symptoms during the attack in our cohort.

In various studies, the response rate to colchicine initiated for prophylaxis in PFAPA disease ranges between 45% and 95.1% [6, 11, 13, 15–18]. In our study, a colchicine response rate of 80.7% was observed, which is consistent with the literature. Konte et al. examined clinical factors predicting colchicine response and reported that the presence of tonsillopharyngitis and aphthous stomatitis is associated with a higher likelihood of colchicine resistance and response to tonsillectomy [14]. Our study revealed that the presence of pharyngitis and arthralgia were the only clinical findings associated with colchicine resistance. However, the presence of exudative tonsillitis, aphthous stomatitis, cervical lymphadenopathy, headache, and arthritis did not aid in predicting the response to colchicine.

PFAPA episodes typically last 3–7 days, most commonly around 4–5 days, with recurrences occurring every 14–60 days, often within 3 to 6 weeks [1, 6, 10, 14, 19]. In our study, although the average interval (28 days) between attacks did not differ between the colchicine-responsive and colchicine-unresponsive groups, the duration of attacks was significantly shorter (3.8 days *vs* 4.1 days, *p* = 0.001) and the annual attack frequency was lower (13.6 vs 16.1, *p* = 0.001) in the colchicine-responsive group than in the colchicine-unresponsive group.

In clinical practice, patients with PFAPA syndrome typically undergo genetic analysis targeting genes associated with monogenic autoinflammatory diseases, such as the *MEFV* gene, which is linked to familial Mediterranean fever (FMF). Previous studies have demonstrated that carrying *MEFV* variants may impact the clinical course and treatment responses in individuals with PFAPA [6, 7, 12, 13, 16, 18, 20–22]. For instance, Pehlivan et al. [12] showed that PFAPA patients with *MEFV* variants exhibited a better response to colchicine than those without *MEFV* variants (66% *vs* 33%, respectively, *p* = 0.003). Furthermore, a 6-month open-label, randomized, controlled study by Butbul et al. [7] showed a decrease in attack frequency in six out of eight patients who were started on colchicine and reported that five of these patients had *MEFV* mutations (heterozygous M694V in 3 and E148Q in 2). A multicenter study conducted in France analyzing 20 children with PFAPA syndrome demonstrated that nine patients responded to colchicine. Among these nine responders, *MEFV* mutations were detected in five patients (heterozygous M694V in 4 and V726A in 1). The study indicated that heterozygous *MEFV* variants were more common in the colchicine responder group but did not reach statistical significance [18]. Gunes et al. [6], in their study involving 400 PFAPA cases, conducted genetic analyses on 231 patients and identified heterozygous *MEFV* gene variants in 57 individuals. The most prevalent mutation was heterozygous M694V, which was observed in 23 (10%) patients. The present study found that the presence of the *MEFV* gene variant was significantly associated with a reduction in attack frequency (*p* = 0.003). Konte et al. [14] detected *MEFV* gene variants in 106 of 131 PFAPA patients (80.9%), with M694V heterozygous being the most common variant. They found that the presence of tonsillopharyngitis, aphthous stomatitis, and a family history of PFAPA were associated with colchicine resistance, whereas those with exon 10 *MEFV* gene mutations were more likely to respond favorably to colchicine. In contrast, Yener et al. [13] evaluated colchicine responsiveness in 157 patients with PFAPA syndrome and concluded that carrying an *MEFV* variant did not influence the response. In our study, *MEFV* gene sequencing was performed on 571 (70.8%) patients. The predominant *MEFV* variants identified were M694V at a frequency of 16.8%, E148Q at 9.1%, V726A at 3.5%, and M680I at 2.6%. Our analysis did not reveal any notable variance in colchicine response between patients harboring *MEFV* variants and those with exon 10 variants. Nevertheless, we observed a notably elevated rate of response to colchicine among patients carrying the *M694V* variant. The high prevalence of *MEFV* variants in patients with PFAPA may be explained at the high carrier rate in the healthy population [23].

The most significant limitation of this study was its retrospective design. However, it is strengthened by its large sample size and multicenter design. Another important limitation is the national focus, which precludes an examination of potential ethnic variations in the findings. The disease course may be influenced by environmental and epigenetic factors over time. Therefore, prospective studies may be more constructive in this regard.

In our study, the presence of pharyngitis or arthralgia and more frequent attacks were found to be independently associated with colchicine unresponsiveness, whereas carrying the M694V variant was the sole factor predicting colchicine responsiveness. In conclusion, genetic predisposition, clinical features, and disease course play critical roles in determining the response to colchicine, the cornerstone prophylactic treatment for PFAPA syndrome. Predicting whether colchicine will be beneficial is crucial for clinicians in managing patients diagnosed with PFAPA, informing families, and determining the timing of treatment options such as tonsillectomy.

## Data Availability

The authors are pleased to share the data upon request.

## Abbreviations

CRP: C-reactive protein
ESR: Erythrocyte sedimentation rate
FMF: Familial Mediterranean fever
MEFV: *Mediterranean FeVer*
PFAPA: Periodic fever, aphthous stomatitis, pharyngitis, and cervical adenitis syndrome
WBC: White blood cell count
